# Supplementary material for: Improved Thermal Anisotropy of Multi-Layer Tungsten Telluride on Silicon Substrate
Source: Nanomaterials (Basel). 2023 Jun 7;13(12):1817. doi: 10.3390/nano13121817 (PMC10302967; doi:10.3390/nano13121817)
Supplement: Supplementary file 1 [file nanomaterials-13-01817-s001.zip › nanomaterials-2430859-supplementary.pdf]

Supporting Information

Improved thermal anisotropy of multi−layer tungsten telluride on silicon substrate

Mengke Fang [a, b], Xiao Liu [a, b], Jinxin Liu [a, b], Yangbo Chen [a], Yue Su [a, b], Yuehua Wei [c], Yuquan Zhou [a], Gang Peng [b], Weiwei Cai [a, d, *], Chuyun Deng [b, *], and Xue-Ao Zhang [a, d, *]

[a] College of Physical Science and Technology, Xiamen University, Xiamen 361005, China; 19820201153527@stu.xmu.edu.cn (M.F.); xiaoliu@stu.xmu.edu.cn (X.L.); jxliu@stu.xmu.edu.cn (J.L.); bobbychen@stu.xmu.edu.cn (Y.C.); 19820190154690@stu.xmu.edu.cn (Y.S.); yqzhou@stu.xmu.edu.cn (Y.Z.)
[b] College of Science, National University of Defense Technology, Changsha 410073, China; penggang@nudt.edu.cn
[c] College of Advanced Interdisciplinary Studies, National University of Defense Technology, Changsha 410073, China; weiyuehua18@nudt.com
[d] Jiujiang Research Institute of Xiamen University, Jiujiang 332105, China;
*Correspondence: wwcai@xmu.edu.cn (W.C.); dengchuyun@nudt.edu.cn (C.D.); and xazhang@xmu.edu.cn (X.Z.)

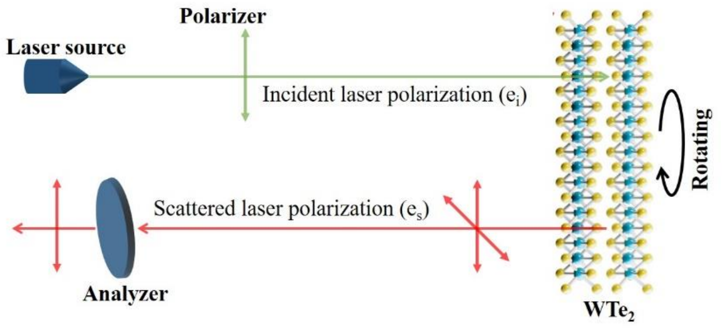

Figure S1. The schematic diagram of polarized−Raman configuration. A polarizer and an analyzer are integrated inside the Raman spectrometer. The laser polarization is fixed along a certain axis by a polarizer and the analyzer direction is set to the same axis, allowing the analysis of the scattered light (e_s) polarized parallel to the incident light polarization (e_i), which corresponds to the parallel polarization configuration (labeled as $e_i \parallel e_s$) [1, 2]. All Raman spectra are acquired with 532nm laser wavelength and 50x objective (N_A=0.55, and the laser spot size is about 616nm).

Angle-dependent Raman spectroscopy of WTe_2 samples

Raman spectroscopy is a common tool to characterize the lattice structure, electrical, optical, and phonon properties of 2D materials [3-5]. Angle−dependent Raman spectroscopy was extensively used

to study anisotropic lattice structures [1, 2, 6]. During the characterization of angle−dependent Raman spectroscopy, we fix the incident laser polarization (e_i) parallel to the scattered laser polarization (e_s) (corresponds to parallel polarization configuration, labeled as $e_i \parallel e_s$) and set θ as the angle between the incident laser polarization (e_i) and the zigzag axis. We place the zigzag axis of WTe_2 sample parallel to the incident laser polarization (that is θ=0°) [1, 2] and the sample could be rotated clockwise from 0° to 360° by a sample stage. The optical anisotropy and the lattice orientation of WTe_2 sample are then investigated by analyzing the variation of Raman intensity with θ. The details of angle−dependent Raman spectroscopy are presented in the illustrations of Figure 1 and Figure S2.

Raman thermometry measurements of WTe_2 samples

Raman thermometry is an important method for investigating the thermal conductivity of 2D materials [7-9]. As the temperature increases, Raman peak frequency will shift towards the lower frequency which means that the Raman peak is redshifted [8]. The thermal conductivity can be obtained from the change in Raman frequency [10]. The thermal conductivity of WTe_2 sample is investigated by Raman thermometry from the following two steps [11-13]. First, the in situ Raman spectra are obtained under a certain temperature range, from which the coefficient between Raman peak frequency and temperature could be extracted. The temperature−dependent in situ Raman spectra are performed with the temperature range from liquid nitrogen to room temperature to avoid damage to sample [11-13]. Second, the sample is heated by increasing the laser power/bias voltage to analyze the Raman peak frequency shift against changing power. Laser power−dependent and bias voltage−dependent in situ Raman spectra are performed with low laser power and bias voltage range at room temperature [11, 13]. Here, based on the different heating sources, Raman thermometry can be divided into laser−heating Raman thermometry (heating the sample with laser power) [12] and electric−heating Raman thermometry (heating the sample with electrical power) [11]. Considering the anisotropic lattice structure of WTe_2, temperature− and laser power/bias voltage−dependent in situ Raman spectra are performed along zigzag and armchair directions, respectively. We obtain the thermal conductivity of WTe_2 sample along zigzag direction by characterizing the in situ Raman spectra, where zigzag axis is aligned with the incident laser polarization (defined as initial 0°). Then, the in situ Raman spectra along the armchair direction are measured by rotating the sample by 90° so that armchair axis is aligned with the incident laser polarization.

The thermal conductivity of suspended WTe_2 flake is measured by laser−heating Raman

thermometry [12]. The in–plane thermal conductivity could be calculated by the equation $\kappa = \chi_T\ (1/2\pi h)\ (\delta\omega/\delta P_A)^{-1}$, where P_A is the absorbed laser power (P_A =AP, A is the absorptivity and P is the incident laser power), δω is the Raman peak position shift caused by the increase of absorbed laser power δP_A, h represents the flake thickness. On the other hand, the thermal conductivity of supported WTe_2 flake is measured by electrical–heating Raman thermometry [11]. Based on the linearly fitting curve of the temperature and the channel power density, we could calculate the in–plane thermal conductivity by the equation $T = T_0 + P/\kappa_{\perp}$, where P is the power density per channel length and T_0 is the temperature when the bias voltage is 0V, $\kappa_{\perp}$ is that along the direction perpendicular to current. Finally, the thermal anisotropy ratio is obtained by comparing the thermal conductivity of different lattice orientations, as presented in the illustrations of Figures 2-4.

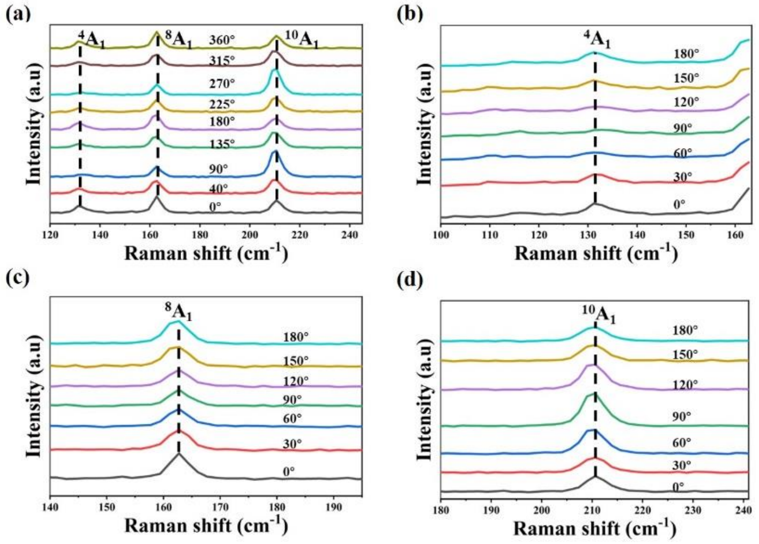

Figure S2. (a-d) The angle–dependent Raman spectra of WTe_2 flake supported on the SiO_2/Si substrate rotated clockwise from 0° to 360° under 532nm laser in the parallel configuration. **(a)** The Raman spectra for three A_1 modes. The enlarged Raman spectra for **(b)** $^4A_1$, **(c)** $^8A_1$ and **(d)** $^{10}A_1$.

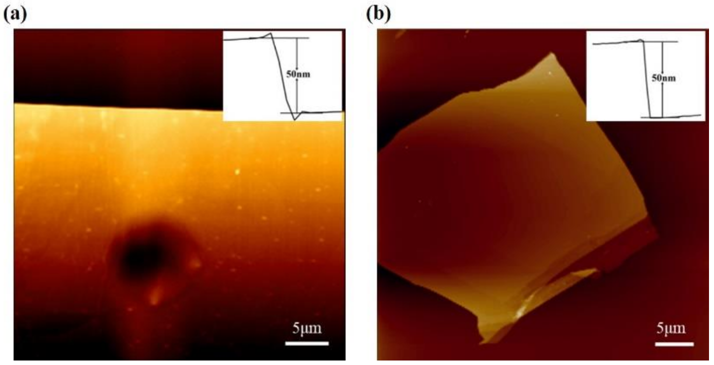

Figure S3. The AFM images of **(a)** suspended and **(b)** supported WTe_2 samples. The insets show

corresponding height profile, indicating that the thicknesses of suspended WTe_2 flake (suspended on silicon substrate with hole diameter of 12μm) and supported WTe_2 flake (supported on SiO_2/Si substrate) are approximately 50nm. Scale bar is 5μm.

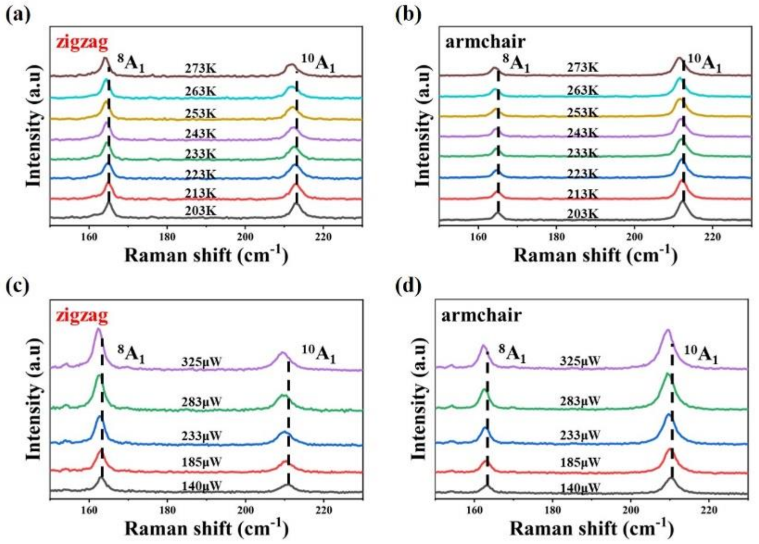

Figure S4. (a, b) Temperature–dependent and **(c, d)** laser power-dependent in situ Raman spectra along zigzag and armchair directions of suspended WTe_2 flake.

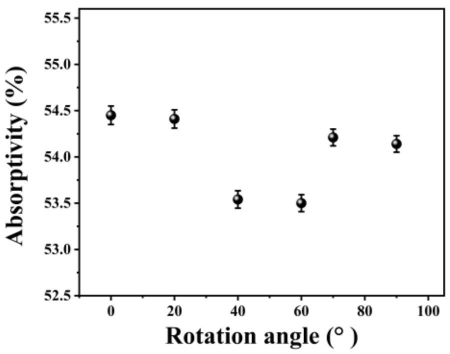

Figure S5. The angle–dependent absorbance plot of WTe_2 flake suspended on silicon substrate with holes (With the diameter of 12μm) rotated clockwise from 0° to 90° at 532nm wavelength. According to the above angle–dependent Raman spectra, it is known that 0° corresponds to zigzag direction and 90° to armchair direction.

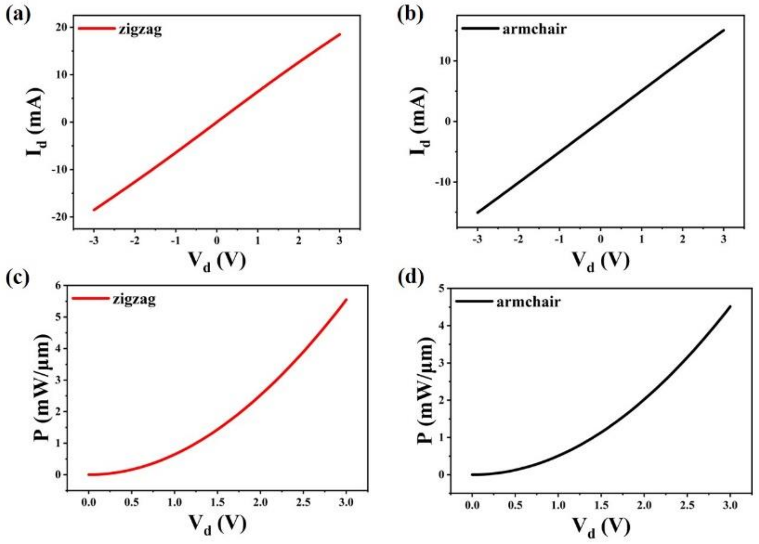

Figure S6. The output IV curves of supported WTe_2 flake along **(a)** zigzag and **(b)** armchair directions. The relationship of electrical power density and bias voltage along **(c)** zigzag and **(d)** armchair for supported WTe_2 flake.

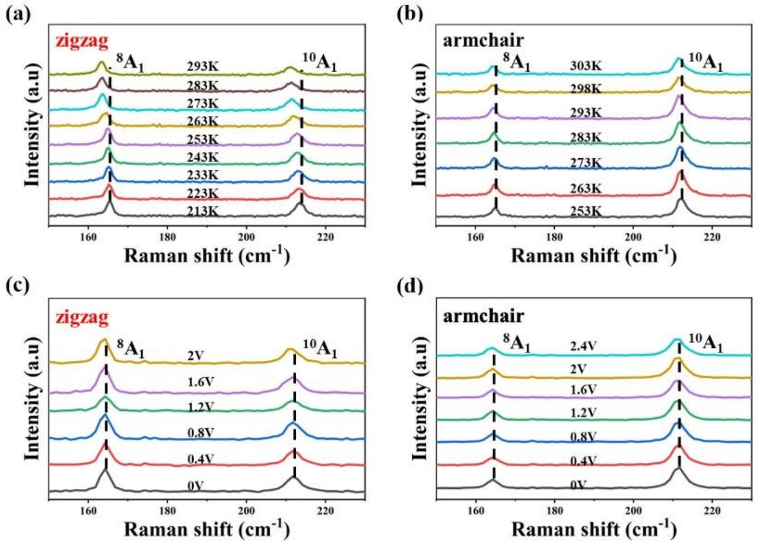

Figure S7. (a, b) Temperature−dependent and **(c, d)** bias voltage−dependent in situ Raman spectra along zigzag and armchair directions of supported WTe_2 flake.

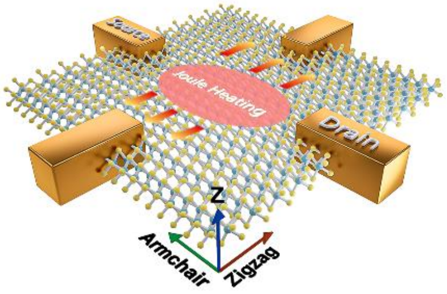

Figure S8. Schematic diagram of in−plane thermal dissipation in WTe_2 device. Joule heat is generated when the current flows along armchair direction of WTe_2 device, which is preferentially dissipated along zigzag direction with greater thermal conductivity.

Table S1. The enhanced thermal anisotropy ratio (κ_{zigzag} / $\kappa_{armchair}$) of BP and WTe_2 caused by substrate coupling. The supported BP sheet [14] and WTe_2 flakes [11, 13] exhibit a significantly higher thermal anisotropy ratio compared to the suspended one, which may be a result of the presence of substrate has affected the thermal conductivity in an uneven manner. Among them, the anisotropy ratio of BP sheet will be improved with the increase of the substrate coupling strength [14], demonstrating that substrate coupling can modulate the thermal anisotropy ratio of anisotropic 2D materials.

Material	$\kappa_{zigzag}/\kappa_{armchair}$	References
Suspended BP sheet	3.1	[14]
Supported BP sheet	4.4	
27nm thick suspended WTe_2 flake	1.1	[13]
30nm thick supported WTe_2 flake	1.6	[11]
50nm thick suspended WTe_2 flake	1.09	This work
50nm thick supported WTe_2 flake	1.89	

References:

1. Ribeiro, H. B.; Pimenta, M. A.; De Matos, C. J.; Moreira, R. L.; Rodin, A. S.; Zapata, J. D.; De Souza, E. A.; Castro Neto, A. H., Unusual angular dependence of the Raman response in black phosphorus. *ACS nano* **2015,** *9* (4), 4270-4276.
2. Zhou, Z.; Cui, Y.; Tan, P.-H.; Liu, X.; Wei, Z., Optical and electrical properties of two-dimensional anisotropic materials. *Journal of Semiconductors* **2019,** *40* (6), 061001.
3. Zhang, X.; Tan, Q.-H.; Wu, J.-B.; Shi, W.; Tan, P.-H., Review on the Raman spectroscopy of different types of layered materials. *Nanoscale* **2016,** *8* (12), 6435-6450.

4. Wu, J. B.; Zhao, H.; Li, Y.; Ohlberg, D.; Shi, W.; Wu, W.; Wang, H.; Tan, P. H., Monolayer molybdenum disulfide nanoribbons with high optical anisotropy. *Advanced Optical Materials* **2016,** *4* (5), 756-762.
5. Wu, J.-B.; Lin, M.-L.; Cong, X.; Liu, H.-N.; Tan, P.-H., Raman spectroscopy of graphene-based materials and its applications in related devices. *Chemical Society Reviews* **2018,** *47* (5), 1822-1873.
6. Song, Q.; Pan, X.; Wang, H.; Zhang, K.; Tan, Q.; Li, P.; Wan, Y.; Wang, Y.; Xu, X.; Lin, M., The in-plane anisotropy of WTe_2 investigated by angle-dependent and polarized Raman spectroscopy. *Scientific reports* **2016,** *6* (1), 1-9.
7. Balandin, A. A.; Ghosh, S.; Bao, W.; Calizo, I.; Teweldebrhan, D.; Miao, F.; Lau, C. N., Superior thermal conductivity of single-layer graphene. *Nano letters* **2008,** *8* (3), 902-907.
8. Yan, R.; Simpson, J. R.; Bertolazzi, S.; Brivio, J.; Watson, M.; Wu, X.; Kis, A.; Luo, T.; Hight Walker, A. R.; Xing, H. G., Thermal conductivity of monolayer molybdenum disulfide obtained from temperature-dependent Raman spectroscopy. *ACS nano* **2014,** *8* (1), 986-993.
9. Peimyoo, N.; Shang, J.; Yang, W.; Wang, Y.; Cong, C.; Yu, T., Thermal conductivity determination of suspended mono-and bilayer WS_2 by Raman spectroscopy. *Nano Research* **2015,** *8*, 1210-1221.
10. Chen, C.-C.; Li, Z.; Shi, L.; Cronin, S. B., Thermal interface conductance across a graphene/hexagonal boron nitride heterojunction. *Applied Physics Letters* **2014,** *104* (8), 081908.
11. Wei, Y.; Deng, C.; Zheng, X.; Chen, Y.; Zhang, X.; Luo, W.; Zhang, Y.; Peng, G.; Liu, J.; Huang, H., Anisotropic in-plane thermal conductivity for multi-layer WTe_2. *Nano Research* **2022,** *15* (1), 401-407.
12. Su, Y.; Deng, C.; Liu, J.; Zheng, X.; Wei, Y.; Chen, Y.; Yu, W.; Guo, X.; Cai, W.; Peng, G., Highly in-plane anisotropy of thermal transport in suspended ternary chalcogenide Ta_2NiS_5. *Nano Research* **2022,** *15* (7), 6601-6606.
13. Chen, Y.; Peng, B.; Cong, C.; Shang, J.; Wu, L.; Yang, W.; Zhou, J.; Yu, P.; Zhang, H.; Wang, Y., In-Plane Anisotropic Thermal Conductivity of Few-Layered Transition Metal Dichalcogenide Td-WTe_2. *Advanced Materials* **2019,** *31* (7), 1804979.
14. Chen, J.; Chen, S.; Gao, Y., Anisotropy enhancement of thermal energy transport in supported black phosphorene. *The journal of physical chemistry letters* **2016,** *7* (13), 2518-2523.
